# Variety Discrimination Power: An Appraisal Index for Loci Combination Screening Applied to Plant Variety Discrimination

**DOI:** 10.3389/fpls.2021.566796

**Published:** 2021-03-18

**Authors:** Yang Yang, Hongli Tian, Rui Wang, Lu Wang, Hongmei Yi, Yawei Liu, Liwen Xu, Yaming Fan, Jiuran Zhao, Fengge Wang

**Affiliations:** Maize Research Center, Beijing Academy of Agriculture and Forestry Sciences (BAAFS), Beijing Key Laboratory of Maize DNA Fingerprinting and Molecular Breeding, Beijing, China

**Keywords:** plant variety discrimination, molecular markers, loci combination screening, simple sequence repeats, single nucleotide polymorphism, variety discrimination power

## Abstract

Molecular marker technology is used widely in plant variety discrimination, molecular breeding, and other fields. To lower the cost of testing and improve the efficiency of data analysis, molecular marker screening is very important. Screening usually involves two phases: the first to control loci quality and the second to reduce loci quantity. To reduce loci quantity, an appraisal index that is very sensitive to a specific scenario is necessary to select loci combinations. In this study, we focused on loci combination screening for plant variety discrimination. A loci combination appraisal index, variety discrimination power (VDP), is proposed, and three statistical methods, probability-based VDP (P-VDP), comparison-based VDP (C-VDP), and ratio-based VDP (R-VDP), are described and compared. The results using the simulated data showed that VDP was sensitive to statistical populations with convergence toward the same variety, and the total probability of discrimination power (TDP) method was effective only for partial populations. R-VDP was more sensitive to statistical populations with convergence toward various varieties than P-VDP and C-VDP, which both had the same sensitivity; TDP was not sensitive at all. With the real data, R-VDP values for sorghum, wheat, maize and rice data begin to show downward tendency when the number of loci is 20, 7, 100, 100 respectively, while in the case of P-VDP and C-VDP (which have the same results), the number is 6, 4, 9, 19 respectively and in the case of TDP, the number is 6, 4, 4, 11 respectively. For the variety threshold setting, R-VDP values of loci combinations with different numbers of loci responded evenly to different thresholds. C-VDP values responded unevenly to different thresholds, and the extent of the response increased as the number of loci decreased. All the methods gave underestimations when data were missing, with systematic errors for TDP, C-VDP, and R-VDP going from smallest to biggest. We concluded that VDP was a better loci combination appraisal index than TDP for plant variety discrimination and the three VDP methods have different applications. We developed the software called VDPtools, which can calculate the values of TDP, P-VDP, C-VDP, and R-VDP. VDPtools is publicly available at https://github.com/caurwx1/VDPtools.git.

## Introduction

Plant breeding materials in the hands of breeders and newly bred varieties in the market have emerged in large amounts along with the development of breeding technology, improvement of industry regulations, and industrialization of seed businesses (Yang et al., 2014, 2020). This has resulted in an increased demand for methods that can distinguish plant varieties for application in molecular plant breeding, new plant variety protection, value for cultivation and use tests, seed market management, and rights-defending or anti-counterfeiting activities of breeders (Varshney et al., 2006; Bonow et al., 2009; Julier et al., 2018). Thanks to the advancement of biology, molecular marker technology is now featured with a short testing period, fast processing of high-throughput samples, and stable results and has become a major mainstream method for plant variety identification (Wang et al., 2018). International Union for the Protection of New Varieties of Plants (UPOV) has raised three models of distinctness, uniformity, and stability (DUS) tests by using molecular marker data. Model 1 is to use functional molecular markers that are linked to morphological characters to predicate the phenotypic data; model 2 is to set up an application model that combines both morphological characters and molecular markers by analyzing the relationship between the two; model 3 is to set up a brand new and independent appraisal system with molecular marker characteristics (International Union for the Protection of New Varieties of Plants, 2011). By raising these models, UPOV confirmed that molecular markers could play a positive role in examining DUS.

Currently, simple sequence repeats (SSR) and single nucleotide polymorphisms (SNP) are the two most frequently used DNA markers. The increased availability of reference genomes of many plant species and the lowering sequencing costs have allowed the discovery of large numbers of SSR and SNP loci (Chen et al., 2014; Zhang et al., 2015; Vieira et al., 2016; Liu et al., 2017). To keep low cost and high throughput of sample processing, it is common to use capillary electrophoresis (e.g., ABI3730 DNA analyzer) as SSR genotyping platforms and Kompetitive Allele-Specific PCR (e.g., LGC SNP Line) as SNP genotyping platforms. However, these platforms usually require the number of loci to be controlled at the order of magnitude from E+1 to E+2, and that is why we need to use the method of molecular marker screening to determine a smaller but highly effective group of markers (Semagn et al., 2014). Molecular marker screening refers to the screening of candidate loci that share a certain type of molecular marker. Such screening usually involves two phases: the first to control loci quality and the second to reduce loci quantity. The traditional method of molecular marker screening for plant variety discrimination is to generate fingerprint data of all candidate loci of representative samples, calculate appraisal indices such as position on the genetic map, genetic diversity, polymorphism information content, and minor allele frequency, and then set the sequence and threshold for the molecular markers based on these indices to decide the final loci combinations (Chakraborty et al., 1988; Yamasaki et al., 2005; Yoon et al., 2007; Kavakiotis et al., 2015). In previous research, the performance of loci combinations was usually appraised by randomly selecting different sizes of SNP panels, calculating the genetic distance matrices of the various panels and then conducting correlation analyses of such matrices (Chen, 2016; Liu et al., 2017). There has not been any systematic description of loci combination appraisal methods. The fast development of molecular biotechnology has made available many recently discovered molecular markers with high quality, which makes it less efficient to screen out a few target loci by simple methods such as ranking or threshold setting. Thus, the traditional method mentioned earlier is sufficient for the first phase of screening (control of loci quality) but not the second phase (reduction of loci quantity, or we may call it “loci combination screening”), which is especially important to lower the cost of acquiring DNA fingerprint data and to improve the efficiency of data analysis.

Loci combination screening aims to optimize the number of qualified loci combinations and screen for combinations fit for specific application scenarios but with fewer loci that collectively provide an equivalent or improved outcome. In previous research, this issue was converted to a response matrix constructed by markers and varieties, and then, some algorithms such as an integer linear programming formulation were proposed to determine the minimum loci combination, but when the response matrix became very large, the computational complexity of the algorithms would rise sharply, eventually leading to no calculation results (Gale et al., 2005; Fujii et al., 2013). In case there is a large number of loci or samples, we can achieve such an aim by using a combinatorial optimization algorithm (Rousselle et al., 2015; International Union for the Protection of New Varieties of Plants, 2018, 2019). In this algorithm, an appraisal index is needed as its fitness function, and the fitness function shall be a converging one. In terms of plant variety discrimination, the appraisal index refers to a calculation method that describes the capability of a loci combination to distinguish different varieties. It works mainly as the fitness function of a combinatorial optimization algorithm, which can be used to screen out targeted loci combinations with fewer loci. In previous research, some calculation methods of fitness function have been mentioned. For example, the standard deviation of the scaled physical distance and genetic distance intervals between selected markers can be used to evaluate SNP panels, but the method requires a lot of genetic background information of loci and has high time complexity (Rousselle et al., 2015). Another example is using the number of varieties showing unique genotypes to evaluate SNP combinations, but the method did not provide a clear definition and statistics for its important parameter, unique genotypes, and has never proved its validity systematically (Du et al., 2019). In this study, we looked for a loci combination appraisal index that was not only suitable for the overall appraisal of loci combinations but also sensitive to plant variety discrimination to act as the fitness function of the algorithm to screen loci combinations. Such an appraisal index would become the base and prelude of further research, such as selecting a subset of markers using an optimal combination algorithm. From the aspect of methodology and computer science, we believe that the appraisal index should: (1) make no reference to the genetic background information of the loci; (2) take no account of linkages or associations between loci; and (3) require relatively low computational complexity, especially as low time complexity and space complexity as possible.

Appraisal indices to meet similar targets have been used in other scientific fields. For example, the Simpson index is an appraisal index for community diversity in ecology and is the first statistical index to use probability theory to describe individual similarity (Simpson, 1949). This index evolved into the Gini–Simpson index, which describes the probability of interspecific encounters in ecology (Lyons and Hutcheson, 1979; Caso and Gil, 1988). Subsequently, the Gini–Simpson index has been applied widely in other scientific fields and has evolved further into various professional appraisal indices that use similar statistical methods (or equations; McIntosh, 1967; Hunter and Gaston, 1988; Dillon et al., 1993; Gorelick, 2006). Another example is the probability of discrimination power (DP), a statistical index initially proposed to describe individual differences using human blood types (Jones, 1972). DP evolved into an appraisal index that describes individual differences using a single genetic marker and is used in grapevine varietal identification and human forensic identification (Tessier et al., 1999; Pitterl et al., 2010; Butler, 2014). Total probability of discrimination power (TDP) is a comprehensive capability appraisal index used to identify individuals by multiple loci. DP indicates the probability of different molecular marker genotypes of two individuals randomly selected in a group, whereas TDP provides a comprehensive appraisal of multiple molecular markers using a multiplication law on the basis of DP (Jones, 1972). Although TDP addresses issues similar to plant variety discrimination, there are two main problems in applying this index for plant variety discrimination. First, TDP is fundamentally a DP based on a single locus and thus is not efficient in appraising the overall performance of a combination of multiple loci. Second, the TDP results quickly approach the maximum value as the number of loci increases, which leads to very subtle differences among TDP results for combinations with loci above a certain number. This tendency makes TDP a non-efficient appraisal index as the fitness function of optimal combination algorithm to screen a subset of loci.

In this study, we defined three variety discrimination power (VDP) statistical methods for plant variety discrimination according to the characteristics of loci combination screening. Simulated data were used to analyze the sensitivity of TDP and the three VDP methods to various types of variety differences. Then, real sorghum, wheat, maize, and rice genotype data were used to verify the effectiveness of these statistical methods on loci combinations with different numbers of loci and determine the universality of VDP for different species and different molecular marker types. Finally, we compared the influence of different variety threshold settings and missing data on these statistical methods. The aim was to transform the convergence of the fitness function for combinatorial optimization algorithms to obtain the very high variety discrimination sensitivity needed for appraisal indices. We found that the constructed VDP appraisal index was very efficient for plant variety discrimination.

## Materials and Methods

### Analysis Methods

#### Variety Discrimination Power

The integrity of multiple loci or loci combinations was appraised using VDP, a simple but effective statistical index, as the criterion to evaluate the capability of each loci combination to discriminate all given plant varieties. VDP uses a statistical method based on the idea that the genotype of a loci combination can be used to identify specific plant varieties. Theoretically, when a certain loci combination can distinguish all given varieties, the VDP of such a loci combination is 1; otherwise, it is 0. If only partial varieties can be distinguished, the VDP shall be a value between 0 and 1, representing a different degree of discrimination capability. Note that VDP is not a binary value. The method uses a statistical inference definition according to genotype differences among all given varieties so that the distinguishing capability of a loci combination can be calculated using simple statistics with no need to study the detailed genetic information of each locus.

Variety discrimination power is calculated in three steps. First, the genotypes of loci combinations are used to determine the difference between two randomly given samples by calculating several different loci, percentage of different loci, or genetic distances (Nei and Roychoudhury, 1974; Nei, 1978). The calculation method is chosen according to the types of molecular markers used or the order of magnitude of loci quantity. Second, the threshold value of variety genotype difference (hereafter referred to as “variety threshold setting”) is defined, and the two given samples are classified as the same variety or not the same variety, according to the difference value calculated in the first step. The variety threshold value of genotype difference is set based on the genetic background of each species (Heckenberger et al., 2002) or by referring to the current standards of the species. Taking plant variety protection as an example, the calculation method of its variety threshold setting can refer to Model 2 raised by UPOV, i.e., “Calibration of threshold levels for molecular characteristics against the minimum distance in traditional characteristics” (Achard et al., 2020). Third, the efficiency of the loci combinations in distinguishing the varieties is evaluated by statistical analysis and normalization of the threshold defining results. For statistical purposes, we tested the following research hypothesis for VDP values of loci combinations: when all given samples belong to different varieties and the loci combination can correctly describe such phenomenon, the statistical value of VDP shall be its theoretical maximum value. The null hypothesis shall then be: when all given samples belong to different varieties, but the loci combination identifies all samples as one variety, the statistical value of VDP shall be its theoretical minimum value. The statistical method used in this step is the key to the VDP and is the focus of this study. We proposed three statistic methods for VDP: probability-based VDP (P-VDP), comparison-based VDP (C-VDP), and ratio-based VDP (R-VDP).

#### Probability-Based Variety Discrimination Power

Probability-based variety discrimination power uses the principles of permutation and combination and calculates the probability of selecting two samples that belong to different varieties after two times of random sampling without replacement. Samples identified as the same variety according to the genotypes of loci combinations and variety threshold results are classified into one repeated group (*t* groups in total); then, groups with the same number of samples are further classified into one category (*n* categories in total). The number of samples in a group is *R*_*i*_, which also indicates the repeated times that the samples are identified as the same variety. P-VDP was calculated as follows:

(1) $$\text{P-VDP}=1-\frac{\sum_{i=1}^{n}\left(C_{R_{i}}^{2}\times C_{T_{i}}^{1}\right)}{C_{\sum_{i=1}^{n}R_{i}\,T_{i}}^{2}}$$

where *n* is the total number of categories, *R*_*i*_ is the number of samples in one group in category *i*, *T*_*i*_ is the number of groups in category *i*, $\sum_{i=1}^{n}T_{i}\,=t$, $C_{R\,i}^{2}$ is the combined number of picking two unordered outcomes from *R*_*i*_ possibilities, $C_{T\,i}^{1}$ is the combined number of picking one unordered outcome from *T*_*i*_ possibilities, $\sum_{i\,=1}^{n}R_{i}\,T_{i}$ is the total number of all samples, and $C_{\sum_{i\,=1}^{n}R_{i}\,T_{i}}^{2}$ is the combined number of picking two unordered outcomes from all samples. *R*_*i*_ is a positive integer ≥1. For example, if *R*_*i*_ = 1, then each group from category *i* contains one sample; if *R*_*i*_ = 2, then each group from category *i* contains two samples.

When a given loci combination has missing genotype data, the variety threshold cannot be used to determine if the samples belong to the same variety, so *R*_*i*_ and *T*_*i*_ cannot be calculated precisely. Therefore, P-VDP will not be effective for evaluating loci combinations with missing data.

#### Comparison-Based Variety Discrimination Power

Comparison-based variety discrimination power uses global traversal to compare two samples and calculates the proportion of pairs of samples out of all pairs compared that were identified as different varieties according to the genotypes of loci combinations and variety threshold defining results. C-VDP was calculated as follows:

(2) $$\text{C-VDP}=\frac{\sum_{i=1}^{m}\sum_{j=1}^{m}d_{i\,j}}{m\,\left(m-1\right)},\left(i\neq j\right)$$

where *m* is the total number of samples and *d*_*ij*_ is the comparison result; *d*_*ij*_ = 1 when samples *i* and *j* are determined as two different varieties; otherwise, *d*_*ij*_ = 0. Note that samples *i* and *j* are always different samples; that is, the value of *i* is never equal to the value of *j*.

#### Ratio-Based Variety Discrimination Power

Ratio-based variety discrimination power uses the principle of Occam’s razor (Thorburn, 1915) and calculates the proportion of the number of groups with samples identified as different varieties according to the genotypes of loci combinations and variety threshold defining results out of the total number of samples. R-VDP was calculated as follows:

(3) $$\text{R-VDP}=\frac{D+\sum_{k=1}^{p}G_{k}}{m}$$

where *D* is the number of groups that contain only one sample, which was not identified with any other sample as the same variety, *p* is the total number of categories in which a group contains two or more samples, *G*_*k*_ is the number of groups in category *k*, and *m* is the total number of samples. *D*+$\sum_{k\,=1}^{p}G_{k}$ is the total number of groups, equivalent to $\sum_{i=1}^{n}T_{i}$ in Eq. 1.

### Data Sources and Processing

Dataset 1 contained simulated data of variety difference degree (VDD). VDD is generally determined by the diversity of varieties in a sample population; thus, statistical populations with different quantities of varieties can be used to represent those with gradient VDD. We simulated genotype data of 10 multiallelic SNP loci of statistical populations with different quantities of varieties. A multiallelic locus means that the locus contains four types of allelic variation. Gradient VDD simulated four arrays of data through arithmetic progression with a single increment variable. Each array comprised 21 statistical populations with a fixed size of 206 samples in a population. The variable referred to the two independent variables of P-VDP, namely the number of samples in one repeated group (*R*_*i*_) and the corresponding number of repeated groups (*T*_*i*_). In the first array, statistical populations with a convergence of multiple samples to a certain variety were simulated by an arithmetic progression of *R*_*i*_; and in the other three arrays, statistical populations with a convergence of multiple samples to multiple varieties were simulated by an arithmetic progression of *T*_*i*_ with fixed *R*_*i*_. The fixed *R*_*i*_ was increased gradually from the second to the fourth array. The specific variable settings of the four arrays of simulated data are shown in Table 1, and the simulated data are given in Supplementary Tables 1–4. Supplementary Table 5 showed real genotype data of sorghum (Casa et al., 2005) and wheat (Hao et al., 2011) samples based on SSR loci, as well as real genotype data of maize (Tian et al., 2015) and rice (Zhao et al., 2010) samples based on SNP loci. To provide qualified genotype data for TDP and the VDP statistic methods, we randomly extracted 20 loci from SSR and 100 loci from SNP with no missing data to form a new set of real genotype data, which we call dataset 2 (Supplementary Table 6). Dataset 2 contained data of the four species as mentioned earlier with 101, 250, 96, and 391 samples. It is possible to discriminate 1.05E+6 or 1.27E+30 varieties with 20 or 100 loci when the allele number of each locus equals 2. However, in dataset 2, the allele number of each SSR locus is always larger than 2, so theoretically speaking, 20 loci from SSR is sufficient to evaluate the variety discrimination rate of the samples even if all the samples belong to different varieties. Dataset 3 contained simulated genotype data derived from dataset 2 with different rates of missing genotype data. Missing data of each sample were created randomly with proportions that increased progressively by 10% applied to the genotype data subsets in dataset 2 (of only sorghum and maize samples), which had no missing data. The rate of missing data was increased gradually from 0 to 50% (Supplementary Tables 7,8).

**TABLE 1:** Variable settings of SNP simulated data of gradient variety difference degree.

No.	Data array 1			Data array 2			Data array 3			Data array 4		
				
	*D*	*R*_*i*_	*T*_*i*_	*D*	*R*_*i*_	*T*_*i*_	*D*	*R*_*i*_	*T*_*i*_	*D*	*R*_*i*_	*T*_*i*_
1	204	2	1	204	2	1	201	5	1	206	10	0
2	196	10	1	196	2	5	196	5	2	196	10	1
3	186	20	1	186	2	10	186	5	4	186	10	2
4	176	30	1	176	2	15	176	5	6	176	10	3
5	166	40	1	166	2	20	166	5	8	166	10	4
6	156	50	1	156	2	25	156	5	10	156	10	5
7	146	60	1	146	2	30	146	5	12	146	10	6
8	136	70	1	136	2	35	136	5	14	136	10	7
9	126	80	1	126	2	40	126	5	16	126	10	8
10	116	90	1	116	2	45	116	5	18	116	10	9
11	106	100	1	106	2	50	106	5	20	106	10	10
12	96	110	1	96	2	55	96	5	22	96	10	11
13	86	120	1	86	2	60	86	5	24	86	10	12
14	76	130	1	76	2	65	76	5	26	76	10	13
15	66	140	1	66	2	70	66	5	28	66	10	14
16	56	150	1	56	2	75	56	5	30	56	10	15
17	46	160	1	46	2	80	46	5	32	46	10	16
18	36	170	1	36	2	85	36	5	34	36	10	17
19	26	180	1	26	2	90	26	5	36	26	10	18
20	16	190	1	16	2	95	16	5	38	16	10	19
21	1	205	1	6	2	100	6	5	40	6	10	20

*D, number of distinct samples; *R*_*i*_, number of samples in one repeated group; and *T*_*i*_, number of repeated groups.*

Targeting at TDP and the three VDP methods, we used dataset 1 and dataset 2 to analyze the different sensitivity of variety discrimination of these methods, dataset 2 (only sorghum and maize samples) to analyze the sensitivity on variety threshold setting, and dataset 3 to analyze the stability in case of missing data. Sensitivity analysis on variety discrimination indicated how sensitive the output value was to the input value of variety difference. Sensitivity analysis on threshold settings evaluated the influence of different variety threshold settings on the output value. Stability analysis on missing data indicated the adaptability of the methods on the general assumption that some genotype data of the given loci combination are missing.

The statistical differences between the analysis results under different conditions were all analyzed by the Jonckheere–Terpstra test, one of the rank-based nonparametric tests by SPSS software (IBM Corp. Released 2017. IBM SPSS Statistics for Windows, Version 25.0. Armonk, NY, United States: IBM Corp.). For the three datasets, the difference between two randomly given samples was calculated by the number of different loci in the first step of the three VDP methods. In the second step, the variety threshold was set as “different variety when number of different loci of samples was ≥1” for sensitivity analysis on variety discrimination and missing data, and “different variety when number of different loci of samples was ≥*M*” (*M* is 1, 2, 3, or 4) for sensitivity analysis on variety threshold setting. We developed the software called VDPtools, which can calculate the values of TDP, P-VDP, C-VDP, and R-VDP. The types of molecular markers supported by the data file include SSR and SNP. Variety threshold setting methods that can be freely changed by users include the number of different loci and the percentage of different loci. VDPtools is publicly available at https://github.com/caurwx1/VDPtools.git.

## Results

### Sensitivity Analysis of Four Loci Combination Appraisal Methods to Simulated Data of Gradient Variety Difference Degree

**FIGURE 1:**
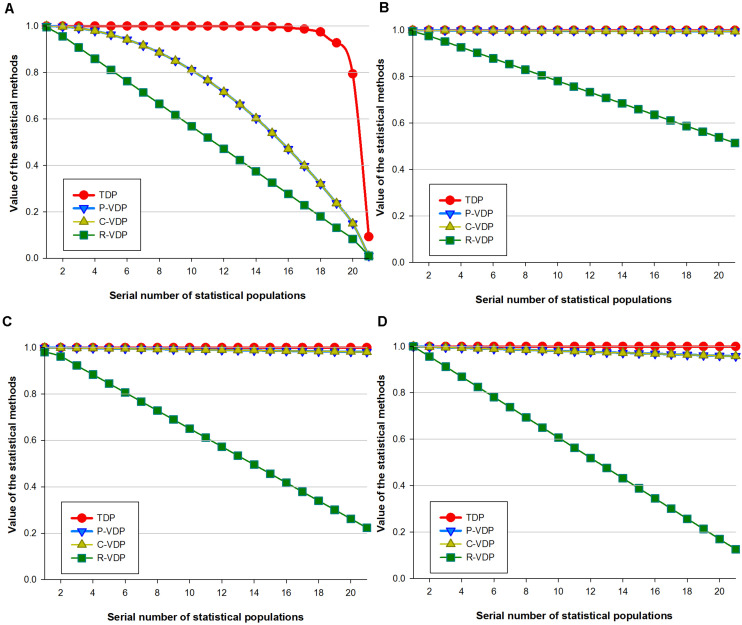
Sensitivity of four loci combination appraisal methods on simulated data of gradient variety difference degree. **(A)** In Data Array 1, repeated samples were those that converged toward the same variety. **(B–D)** In Data Array 2, 3, and 4, repeated samples converged toward various varieties but with decreasing numbers of repeated groups.

Statistical populations were used to simulate gradient VDD to verify the variety discrimination sensitivity of the appraisal methods with different loci combinations. The results for the four simulated data arrays are shown in Figure 1 and Supplementary Table 9. For all the simulated data, the proportion of repeated samples in the group shall grow with the sequence number of the calculated groups (i.e., the value of *x*-axis), so theoretically, the calculation results (i.e., the value of *y*-axis) of an appraisal method shall keep going down. The more even and remarkable such downward tendency is, the more sensitive the appraisal method is in terms of variety discrimination. When the trend of the appraisal results changes in a linear regression model, the slope rate of a univariate linear equation can be used to estimate variety discrimination sensitivity. In Data Array 1, repeated samples were those that converged toward the same variety. Figure 1A shows that with the rise of the proportion of repeated samples, the differences between the TDP values and 1 dropped very gradually from level E-10 to E-3 for the first 16 statistical populations, and the TDP values of the next four statistical populations (17–20) dropped dramatically from 0.987 to 0.794, then to 0.09 for the last population. The estimated VDP values of all three methods dropped evenly from 1 to 0. The P-VDP values were equivalent to the C-VDP values, with the trend lines of both close to a univariate quadratic linear equation; the trend lines of R-VDP sloped down as a univariate linear equation with a slope rate of -0.0487. These results show that for Data Array 1, TDP had low and uneven variety discrimination sensitivity, whereas the three VDP methods all had a high sensitivity. In Data Array 2, repeated samples were those that converged toward various varieties. Figure 1B shows that with the rise of the proportion of repeated samples, all four methods showed a univariate linear equation trend with increasing slope rates of -0.0242, -0.0002, -0.0002, and -7E-11 and minimum estimated values of 0.515, 0.995, 0.995, and 0.999999993 for R-VDP, P-VDP, C-VDP, and TDP, respectively. For Data Array 2, R-VDP had the highest variety discrimination sensitivity, P-VDP and C-VDP had very low sensitivity, and TDP showed no sensitivity. Data Arrays 3 and 4 had the same type of repeated samples as Data Array 2 but with decreasing numbers of repeated groups. Figures 1C,D show that R-VDP had decreasing slope rates of -0.0386 and -0.0437 and minimum estimated values of 0.223 and 0.126 for Data Arrays 3 and 4, respectively. The estimated values of P-VDP and C-VDP were equal, with slightly decreasing slope rates of -0.0009 and -0.0021 and minimum values of 0.981 and 0.957 for the two arrays. TDP maintained horizontal slope rates of -5E-8 and -5E-6 and minimum values of 0.999998 and 0.99990, respectively. The Jonckheere–Terpstra tests showed statistically significant differences in estimated values between different appraisal methods, and the *p*-values of Figures 1A–D were 9.11E-8, 1.19E-18, 1.22E-18, and 1.31E-15, respectively. These results show that for Data Arrays 3 and 4, R-VDP had the highest variety discrimination sensitivity and was evenly affected by different degrees of convergence; that is, the sensitivity increased when the degree of convergence increased. P-VDP and C-VDP showed slowly increasing sensitivity and were slightly affected by different degrees of convergence, and TDP showed an increasing sensitivity trend and was little affected by different degrees of convergence.

### Sensitivity Analysis of Four Loci Combination Appraisal Methods on Genotype Data of Different Plant Species, Different Molecular Marker Types, and Different Numbers of Loci

**FIGURE 2:**
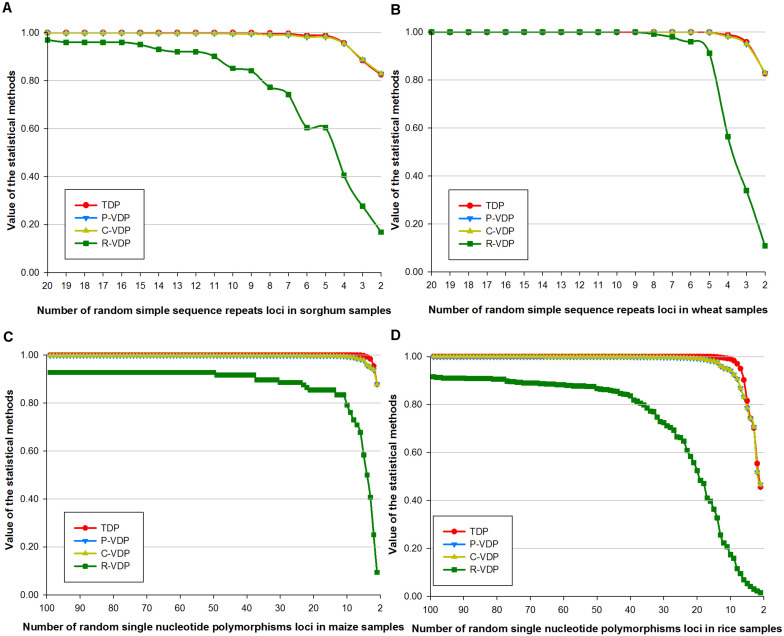
Sensitivity of four loci combination appraisal methods on real data of gradient loci numbers. Real data contained genotype data of **(A)** sorghum samples based on SSR loci, **(B)** wheat samples base on SSR loci, **(C)** maize samples based on SNP loci, and **(D)** rice samples base on SNP loci.

For a certain fixed sample population, the capacity of a loci combination to discriminate different varieties weakens when the number of its loci reduces. Therefore, data of loci combinations with different numbers of loci can also be used to test the variety discrimination sensitivity of the different appraisal methods. To highlight the relationship mentioned earlier between loci combination’s discrimination capacity and its loci number, we used the four groups of real data in dataset 2 as four fixed sample populations and gradually reduced the number of loci in them by nested inclusion. In this way, we got four groups of gradient data. We then calculated the estimated values of the four groups of data by the four appraisal methods and compared their changing tendency to evaluate the sensitivity of the four methods, as shown in Figure 2. The *X*-axis in Figure 2 represents the different number of loci, and the *Y*-axis represents the values calculated by the four appraisal methods. Variety threshold setting has a direct influence on the analysis results, so the thresholds of different species were preset uniformly as “different variety when number of different loci of samples was ≥1,” to highlight and compare the different trends shown when we use different appraisal methods with varied statistical principles to evaluate data of different species. The more remarkable the downward tendency is, the more sensitive the appraisal method is. Data of sorghum (Figure 2A) and maize (Figure 2C) show great similarity: when the R-VDP line drops dramatically in an arc to 0.1, lines of the other three methods stay stable for a long time and then drop slightly to 0.8. For wheat data (Figure 2B), when the R-VDP line stays stable, the lines of the other three methods also stay stable; when the R-VDP line drops quickly to 0.1, the lines of the other three methods drop slowly to 0.8. For rice data (Figure 2D), when the R-VDP line drops from 0.92 to 0.01 in slope, P-VDP and C-VDP lines drop to 0.46 in an arc, and the TDP line drops to 0.45 in an arc after keeping stable (>0.99) for a while. The sensitivity level of each method is measured by order of magnitude of the difference between 1 (the theoretical maximum value) and the calculated value. The bigger the order of magnitude is, the more sensitive the method is and vice versa, as shown in Supplementary Table 10. When the order of magnitude reaches E-2, we can see a trend of changes in Figure 2, so we use E-2 as the threshold setting value that triggers a turning point when using the four methods to analyze the data of different species: TDP lines for sorghum, wheat, maize, and rice data begin to show downward tendency when the numbers of loci are 6, 4, 4, and 11, respectively, whereas in the case of P-VDP and C-VDP (which have the same results), the numbers are 6, 4, 9, and 19, respectively, and in the case of R-VDP, the numbers are 20, 7, 100, and 100, respectively. It can be concluded that the R-VDP line drops first, followed by P-VDP and C-VDP lines, and then the TDP line at the last. What is more, there is a common rule among the four species: when the order of magnitude of the sensitivity level of TDP reaches E-4, that of R-VDP can reach E-1 (with that in the case of wheat reaches E-2 as an exception), and those of P-VDP and C-VDP stay in between. The Jonckheere–Terpstra tests of sorghum, maize, and rice showed statistically remarkable differences between the four appraisal methods, with a *p*-value of 7.79E-9, 2.19E-13, and 7.36E-10, respectively, whereas the Jonckheere–Terpstra test of wheat showed the opposite, with a *p*-value of 0.39. In all cases, R-VDP had the greatest sensitivity, followed by C-VDP, P-VDP, and TDP.

### Relationship Between Probability-Based Variety Discrimination Power and Comparison-Based Variety Discrimination Power

The results in section “*Sensitivity analysis of four loci combination appraisal methods to simulated data of gradient variety difference degree”* and “*Sensitivity analysis of four loci combination appraisal methods on genotype data of different plant species, different molecular marker types, and different numbers of loci”* showed that P-VDP and C-VDP produced the same results despite the different equations they used. To explain these results, the equation for C-VDP was examined using comparison rules to help clarify the relationship between these two methods.

In practical applications, a “discrimination chain” often occurs when judging if two samples belong to the same variety. For example, after random selection and comparison, if samples A and B are identified as the same variety, meanwhile samples B and C are also identified as the same variety, then it stands to reason that samples A and C can be classified as the same variety. If comparisons of any two of all the samples show the same “discrimination chain” characteristic, C-VDP can be converted to calculate the number of pairs of samples that are identified as the same variety as follows:

(4) $$\text{C-VDP}=1-\frac{2\,\sum_{i=1}^{n}\left(C_{R_{i}}^{2}\times C_{T_{i}}^{1}\right)}{m\,\left(m-1\right)}$$

where *m* is the total number of samples, *n* is the total number of categories, *R*_*i*_ is the number of samples in one group in category *i*, *T*_*i*_ is the number of groups in category *i*, $C_{R\,i}^{2}$ is the combined number of picking two unordered outcomes from *R*_*i*_ possibilities, and $C_{T\,i}^{1}$ is the combined number of picking one unordered outcome from *T*_*i*_ possibilities. In Eq. 1, $\sum_{i=1}^{n}R_{i}\,T_{i}$ is the multiply accumulated result of *R*_*i*_ and *T*_*i*_, equaling *m* the total number of all samples. Thus, it can be deduced that Eq. 1 is equivalent to Eq. 4, so C-VDP is equivalent to P-VDP when a “discrimination chain” occurs in sample comparisons.

Loci combinations with no missing genotype data were picked randomly from dataset 2 to verify the relationship between the comparison results of any two samples and the “discrimination chain,” taking the calculation of VDD by the number of different loci as an example. When samples perfectly matched the variety threshold, that is, when the number of different loci was 0, the samples were defined as the same variety. When samples only partially match the variety threshold, that is, when the number of different loci was less than *L* (L > 1), the samples can also be defined as the same variety. Thus, when the variety threshold was set as “perfect match,” the comparison results of any two samples followed the “discrimination chain,” rendering C-VDP equivalent to P-VDP, but when the variety threshold was set as “partial match,” some of the comparison results did not follow the “discrimination chain” because of the inconformity of different loci within the threshold, rendering C-VDP not equivalent to P-VDP. For P-VDP, samples are identified as “the same variety,” so when there is a “partial match,” there will be samples identified as “the same variety” that do not conform with the “discrimination chain”; therefore, the variety threshold of P-VDP must be set as “perfect match.”

### Influence of Different Variety Thresholds on Different Marker Genotype Data Used to Evaluate Variety Discrimination Power

**FIGURE 3:**
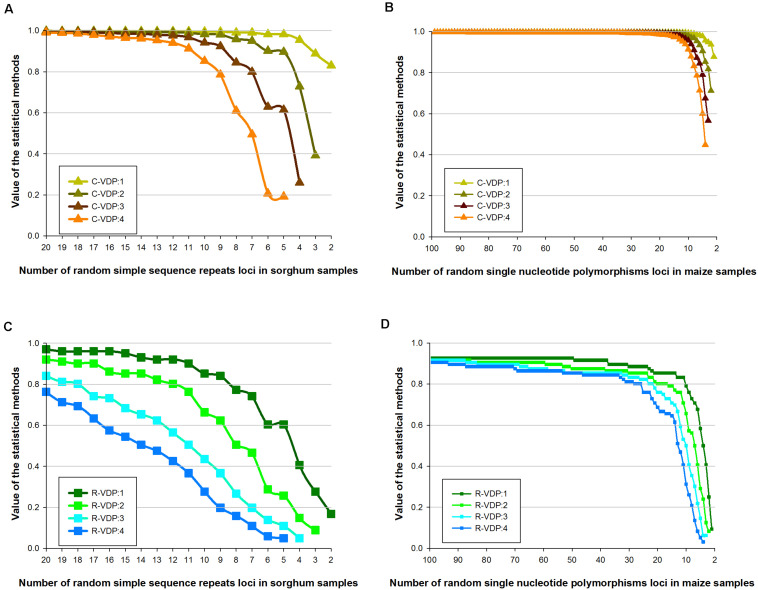
Influence of different variety thresholds on estimated C-VDP and R-VDP values. Genotype data of sorghum samples based on SSR loci is used to evaluate the influence of different variety thresholds on **(A)** C-VDP and **(C)** R-VDP. Genotype data of maize samples based on SNP loci is used to evaluate the influence of different variety thresholds on **(B)** C-VDP and **(D)** R-VDP. Suffix number after C-VDP or R-VDP indicates the threshold setting. For example, C-VDP:1 means the variety threshold of C-VDP was set as “different variety when number of different loci of samples was ≥1.”

According to the definition of DP, the genotype frequency used in TDP makes the situation equivalent to that when the variety threshold is set as a “perfect match.” Because, for P-VDP, the samples are identified as “the same variety,” the variety threshold must be set as “perfect match.” Therefore, the variety threshold parameter influences only the output values of C-VDP and R-VDP, so this setting was evaluated for C-VDP and R-VDP using dataset 2 (of only sorghum and maize samples). The sensitive zone of the variety threshold setting was determined by calculating the difference in VDP values on the same number of loci with adjacent threshold settings and then comparing the differences with different numbers of loci. As shown in Figure 3, lines of four different colors represent the influence of four threshold settings on C-VDP or R-VDP. The titles (i.e., C-VDP:M) referred to “different variety when the number of different loci of samples was ≥*M* (*M* is 1, 2, 3, or 4).” For sorghum data, Figures 3A,C showed that change of variety threshold value could have a dramatic influence on C-VDP in partial range and even influence on R-VDP in full range. Differences among C-VDP values calculated with different threshold settings at more than 10 loci were limited within 0.1, and differences among C-VDP values at less than 10 loci were up to 0.8. An increase of one locus in the minimum value of variety threshold value could bring a decrease of 0.15 in the R-VDP value. For maize data, Figures 3B,D showed that change of variety threshold value could have a dramatic influence on both C-VDP and R-VDP in partial range. When analyzing the different values of a certain statistical method with the same number of loci but different threshold values, we found that: for C-VDP, the difference was less than 0.01 when the number of loci is larger than 20, less than 0.1 when the number of loci is between 10 and 20, and 0.5 at a maximum when the number of loci is smaller than 10; for R-VDP, the difference was less than 0.1 when the number of loci is larger than 27, and 0.6 at maximum when the number of loci is smaller than 26. The threshold setting thus has an influence on VDP values to an extent; however, the Pearson correlation coefficient between values calculated by the same statistical method for the same dataset under different threshold settings is always larger than 0.9. These results showed that the output value of R-VDP was more influenced by the variety threshold than the output value of C-VDP, and the same statistical method showed similar trends on different varieties or different molecular markers.

### Stability Analysis of Three Loci Combination Appraisal Methods on Datasets With Missing Data

**FIGURE 4:**
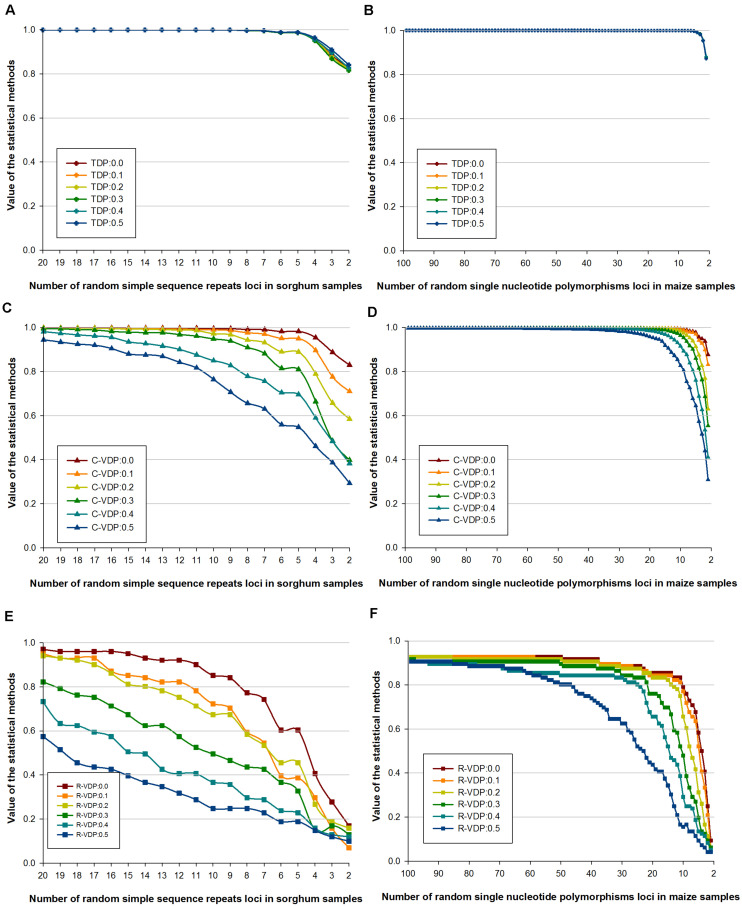
Influence of different missing data rates on the stability of three loci combination appraisal methods. Genotype data of sorghum samples based on SSR loci is used to evaluate the influence of different missing data rates on the stability of **(A)** TDP, **(C)** C-VDP, and **(E)** R-VDP. Genotype data of maize samples based on SNP loci is used to evaluate the influence of different missing data rates on the stability of **(B)** TDP, **(D)** C-VDP, and **(F)** R-VDP. Suffix number after TDP, C-VDP, or R-VDP indicates the missing rate of a dataset. For example, TDP:0.5 means a dataset with missing data rates of 50% was used to estimate the stability of TDP.

Genotype data with missing loci combinations frequently occur in molecular tests, so for practical application, the stability of different appraisal methods on datasets with missing data should be determined. We used dataset 2 (of only sorghum and maize samples) and dataset 3 with missing data rates of 10, 20, 30, 40, and 50%, which is derived from dataset 2, to estimate the stability of TDP, C-VDP, and R-VDP in case of missing data. The stability of each appraisal method in case of missing data was measured using the correlation coefficient between the value for complete data and the values for different missing data rates; a small coefficient indicates low stability and vice versa. For TDP, the values for both molecular markers overlapped for all the datasets tested, as shown in Figures 4A,B. The correlation coefficients for the SSR and SNP loci with 50 and 0% missing data rates were 0.9973 and 0.9999, respectively, which indicated TDP was remarkably stable in missing data and different numbers of loci. For C-VDP, the values for both molecular markers for the datasets with missing data rates of 30% or less and loci numbers more than 10 were similar, which indicated C-VDP had high stability within this scope, as shown in Figures 4C,D. However, the correlation coefficients for SSR and SNP loci with 50 and 0% missing data rates were 0.8091 and 0.8994, respectively, and the average differences of C-VDP values were 0.0491 and 0.0091, respectively, which indicated C-VDP had medium-level stability when missing data rates were more than 30%, and loci numbers were less than 10. For R-VDP, the values for both molecular markers at the different missing data rates had very little overlap, and the differences were greater than they were for C-VDP, as shown in Figures 4E,F. The average differences were 0.0934 and 0.0382 for the SSR and SNP loci, respectively. The correlation coefficients between SSR loci were 0.9804 with 10 and 0% missing data and 0.8375 with 50 and 0% missing data. For the SNP loci, the correlation coefficients were 0.9973 with 10 and 0% missing data and 0.7577 with 50 and 0% missing data, which indicated that R-VDP was very unstable in missing data with all the datasets tested. Overall, these results showed that SSR loci had lower stability in missing data than SNP loci, and TDP was the most stable, followed by C-VDP and R-VDP, which was the most unstable.

## Discussion

### Deduction of and Comparison Between Different Statistic Methods of Loci Combination Appraisal Indices

In the individual discrimination rules of human forensic identification, DP and TDP are used to evaluate the capability of a genetic marker system to discriminate unrelated individuals. Molecular marker screening for plant variety discrimination potentially could use TDP to evaluate loci combinations. However, our results showed that TDP had very low sensitivity to discriminate plant variety differences and thus cannot be used to screen loci combinations. The main aim of screening is to find loci combinations which contain loci with orders of magnitude from E+1 to E+2 to replace those which contain loci with orders of magnitude from E+3 to E+4. To do this, a new index is needed to evaluate the performance of loci combinations to help optimize them. We proposed VDP as an appropriate appraisal index for loci combination screening for plant variety discrimination. The index uses combinations of multiple loci to calculate the performance of each loci combination in every sample. The results showed that VDP had high sensitivity to plant variety differences, which confirmed VDP as the fitness function of optimal combination algorithm to screen a subset of loci for plant variety discrimination.

Genotype data of loci combinations have two dimensions, samples and loci. We proposed three statistical methods of VDP to analyze the data specifically from the dimension of samples. P-VDP was based on probability theory and the most rigorous method in a mathematic sense. Although P-VDP shares a similar mathematic principle with the Gini–Simpson index (Caso and Gil, 1988) or DP, it uses combinations of multiple loci as the subjects of statistics and hence has higher variety discrimination sensitivity than TDP, an index derived from DP. To transform plant variety discrimination into a mathematic issue, P-VDP requires two criteria: the variety threshold must be set as a “perfect match,” and no genotype data are missing in any sample. In practice, these assumptions become major limitations. C-VDP was based on computer graph theory. Although the analysis results and deduced equation for C-VDP showed it was equivalent to P-VDP under the same assumptions, C-VDP can still be used when these assumptions are not met. Thus, C-VDP breaks the limitations of P-VDP and can be used in practical applications to replace P-VDP partially. However, our analysis showed that both these statistical methods lacked effective variety discrimination sensitivity for molecular marker screening of populations in which samples converged to multiple varieties. R-VDP was based on the principle of parsimony, which simplified the calculation and modified the equation with the diversity of convergence varieties. Our results showed that R-VDP had the highest variety discrimination sensitivity but was highly sensitive to missing data. Thus, we propose using P-VDP and C-VDP when variety discrimination sensitivity is not essential and using R-VDP when variety discrimination sensitivity is essential.

### Variety Discrimination Power as an Appraisal Index Compatible With Different Molecular Markers and Species and Adaptable to Diversified Appraisal Demands

Variety discrimination power, as a broad concept of loci combination appraisal indices, can be applied to many fields. First, the types of molecular markers of loci combinations used in VDP appraisal need not be limited to the SSR and SNP codominant molecular markers listed in section “*Sensitivity analysis of four loci combination appraisal methods on genotype data of different plant species, different molecular marker types, and different numbers of loci*.” The method to calculate VDD can be adjusted according to the type of molecular markers adopted so that VDP can be adapted for loci combinations with either dominant or codominant molecular markers. Second, the samples evaluated by VDP need not be limited to the four plant species (sorghum, wheat, maize, and rice) discussed in section “*Sensitivity analysis of four loci combination appraisal methods on genotype data of different plant species, different molecular marker types, and different numbers of loci*.” Variety thresholds can be set according to the plant species of interest, making VDP applicable to all plant species with various differences after breed improvement by a human. Third, the samples evaluated by VDP need not be limited to any particular type. Samples can be from a single plant or multiple plants or from inbred lines or hybrids, and VDP could still be used to evaluate their loci combinations. The type of samples will influence only the representativeness of the analysis results so that VDP can be adapted to population materials. Fourth, VDP is not limited to plant variety discrimination. VDP also could be used to evaluate loci combinations for diversity evaluation of germplasm resources, specificity appraisal by DUS tests in variety protection, and background selection in molecular breeding. In terms of DUS tests for variety protection, it was mentioned in UPOV’s document that “discrimination power of the method” is one of “important considerations for choosing DNA profiling methods that generate high-quality molecular data” and that discriminative capacity is the primary criterion to determine a DNA marker set (International Union for the Protection of New Varieties of Plants, 2020). In this sense, VDP can provide theoretical reference and methodological basis for the research, as mentioned earlier.

### Variety Threshold Setting and Missing Data as Two Main Factors for Estimated Variety Discrimination Power Values

The variety threshold setting influences estimated VDP values. The genetic background of different plant species can be so different than the threshold to reflect differences between varieties should be set according to specific situations. We listed a variety of threshold settings as a separate step in the calculation of VDP and analyzed the influence of different threshold settings on VDP evaluation. P-VDP, by definition, can only have a threshold setting of “perfect match,” so the setting does not influence the estimated VDP values. For C-VDP and R-VDP, the threshold settings are adjustable so that they can influence the VDP values. Generally, for different numbers of loci, the higher the number of loci is, the lower the VDP value is. The setting has the smallest influence on values closest to the maximum and minimum values and the biggest influence on values in the middle level. Therefore, the variety threshold setting is an important open parameter for VDP because it can greatly influence the estimated VDP values. The efficiency of loci combination screening can be improved by adjusting this parameter.

Missing data also can influence estimated VDP values. In practical applications, missing genotype data of loci combinations commonly occur. Only overlapping loci with no missing data in two samples can be used to calculate the variety difference between the samples, so biased estimates of VDP values can occur when data are missing. We analyzed the influences of missing data on the three VDP methods. By definition, P-VDP cannot be used for loci combinations with missing data. For C-VDP and R-VDP, the subjects were samples of “different varieties” or samples with a variety difference that was bigger than the variety threshold value. Although blocking loci with missing data may cause the actual variety difference to be bigger than the variety threshold value and the actual number of samples of “different varieties” to be greater than the statistic number, this system error will lead only to underestimation of the results but will not influence the threshold settings of samples already included in the statistic number. That is to say, blocking loci with missing data will cause a type II error. The results in section “*Stability analysis of three loci combination appraisal methods on datasets with missing data”* confirmed that the bigger the missing data rate is, the bigger the system error is. Therefore, missing data are the main factor for system error in VDP. The missing data rate can be controlled within a certain error tolerance degree according to the actual need.

## Conclusion

To meet the requirements of molecular marker screening in the second phase of plant variety discrimination, we proposed VDP as a new appraisal index for loci combination screening, defined three statistic methods of VDP, and verified the effectiveness of the appraisal index and the three methods by variety discrimination sensitivity analysis with three types of datasets. Based on results from the sensitivity analysis, the most sensitive statistical method was R-VDP, followed by C-VDP, P-VDP, and TDP. When different variety threshold settings were used, R-VDP had a greater sensitivity compared with C-VDP. Finally, R-VDP was the most sensitive method to missing data, followed by C-VDP and TDP. Although VDP was relatively sensitive to the variety threshold setting and missing data, it was generally more suitable than TDP as the fitness function of optimal combination algorithm to screen a subset of loci for plant variety discrimination. The three statistic methods of VDP can be applied as follows: R-VDP for minimal missing data and high requirement for variety discrimination sensitivity; C-VDP for uncontrollable missing data and low requirement for variety discrimination sensitivity; and P-VDP for theory deduction from the VDP method.

## Data Availability Statement

The original contributions presented in the study are included in the article/Supplementary Material, further inquiries can be directed to the corresponding author/s.

## Author Contributions

YY, HT, JZ, and FW conceived the original research plans, drafted the manuscript, and developed the computational method applied in this study. RW, LW, and YF produced the experimental data and performed all bioinformatics analyses. HY, YL, and LX suggested improvements to the method construction and software operation. All authors have read and approved the final manuscript.

## Conflict of Interest

The authors declare that the research was conducted in the absence of any commercial or financial relationships that could be construed as a potential conflict of interest.

## Supplementary Material

Supplementary Table 1 Simulated SNP data array 1 of gradient variety difference degree.

Supplementary Table 2 Simulated SNP data array 2 of gradient variety difference degree.

Supplementary Table 3 Simulated SNP data array 3 of gradient variety difference degree.

Supplementary Table 4 Simulated SNP data array 4 of gradient variety difference degree.

Supplementary Table 5 Raw data of sorghum, wheat, maize and rice.

Supplementary Table 6 Randomly selected loci genotype data with no missing data.

Supplementary Table 7 SSR genotyping data of sorghum samples with gradient missing rate.

Supplementary Table 8 SNP genotyping data of maize samples with gradient missing rate.

Supplementary Table 9 Calculation results of four loci combination appraisal methods on simulated data of gradient variety difference degree.

Supplementary Table 10 Sensitivity of four loci combination appraisal methods on real data of gradient loci numbers.

## Abbreviations

PVP: plant variety protection
VCU: value for cultivation and use
UPOV: International Union for the Protection of New Varieties of Plants
SSR: simple sequence repeats
SNP: single nucleotide polymorphisms
DP: probability of discrimination power
TDP: total probability of discrimination power
VDP: variety discrimination power
P-VDP: probability-based variety discrimination power
C-VDP: comparison-based variety discrimination power
R-VDP: ratio-based variety discrimination power
VDD: variety difference degree.
